# The Effort‐Based Forage Task: An Ethological Behavioral Test for Assessing Motivation and Apathy‐Related Behavior in Mice

**DOI:** 10.1002/cpz1.70195

**Published:** 2025-09-03

**Authors:** Eleanor W. Grayson, Foteini Xeni, Caterina Marangoni, Megan G. Jackson

**Affiliations:** ^1^ School of Physiology, Pharmacology and Neuroscience University of Bristol Bristol United Kingdom

**Keywords:** ethological, innate, mouse, motivation, pharmacology

## Abstract

Apathy and other disorders of motivation represent a significant clinical problem but do not have an agreed treatment approach. The use of translational animal models could facilitate drug development and advance treatment approach. The effort‐based forage task provides a readout of motivational state in mouse models based on their intrinsic drive to forage for nesting material. In this task the mouse is placed in an arena composed of an enclosed home area and a foraging area joined via a tube. Throughout the session the mouse can freely choose to traverse the tube to reach the forage area, obtain nesting material, and shuttle it back to the home area. The nesting material requires effort to obtain and is pulled through apertures in a custom designed nesting material box. The amount of nesting material foraged provides a readout of motivational state, where a deficit in foraging indicates a reduction in motivation. The task does not require physiological (food/water) restriction to motivate the animal to perform in the task, and it does not require training beyond initial habituation to the task environment. The task has been used in behavioral phenotyping of disease models and has been used to test the effects of a wide range of pharmacological manipulations on motivational state. The task environment can be altered to test additional behavioral components that contribute to motivational deficit including effort‐based modulation of behavior and affective reactivity. Overall, the task provides a rapid, translationally relevant method for understanding changes in motivated behavior independent of physiological restriction at the preclinical level. © 2025 The Author(s). Current Protocols published by Wiley Periodicals LLC.

**Support Protocol**: Animal husbandry and arena set up

**Basic Protocol**: Habituation and acute pharmacological manipulation

**Alternate Protocol 1**: The effort curve paradigm

**Alternate Protocol 2**: Affective reactivity test

## INTRODUCTION

Disorders of motivation such as apathy syndrome are highly prevalent across a wide range of neurological and neurodegenerative disorders and can profoundly impact quality of life and disease progression (Chase, 2011; Kos et al., 2016; Malpetti et al., 2021; Zhu et al., 2019). Apathy can occur both as a symptom of a wider disease and as a distinct clinical syndrome. Despite its clear clinical importance, apathy currently has no agreed treatment approach (Sansone & Sansone, 2010; Theleritis et al., 2019) and existing treatments for the underlying conditions with which apathy occurs often fail to effectively address this symptom. Use of animal models that accurately capture behavioral domains relevant to apathy could provide insight into treatment targets and relevant underlying neurobiology that may advance our treatment approach.

Tests relating to motivation to exert effort to obtain rewards are commonly used to assess the behavioral/auto‐activational domain of apathy in both humans and rodents. At the rodent level, this behavior is assessed using operant conditioning‐based paradigms, such as the effort for reward task or progressive ratio task (Marangoni et al., 2023; Richardson & Roberts, 1996; Salamone et al., 1994). In these tasks, the rodent is trained over a period of weeks to months to make an effortful number of instrumental responses (i.e., lever presses, nose pokes) to receive a palatable food or liquid reward. In the case of the progressive ratio task, the rodent must make increasing numbers of responses to obtain a reward. The point at which the rodent decides that the effort requirement is too high and stops responding is called the breakpoint. A higher breakpoint reflects a greater motivational state. In the effort for reward task, the rodent is presented with two options. They can make an effortful but fixed number of responses to receive a high value reward, or consume freely available lab chow, representing a low effort, low value option. The choices the rodent makes across the session provides a readout of their motivational state, with a greater number of high effort trials completed reflecting greater levels of motivation. These tasks have been used to provide valuable insight into the role of the mesolimbic dopaminergic system in motivated behavior and the mechanisms underlying a range of clinically used dopaminergic drugs (Nunes et al., 2013; Salamone et al., 2018). However, there are some limitations associated with the use of food‐motivated conditioning methods that require attention.

Food or water restriction is necessary to motivate the rodent to produce robust and reliable responses in these tasks. However, the use of food/water restriction may profoundly alter normal behavior and physiology, impacting the translatability of findings to motivated behavior in a non‐restricted context (Bubenik et al., 1992). Indeed, human equivalent tasks such as the effort expenditure for reward task (EEfRT) (Treadway et al., 2009) are not driven by physiological restriction, raising questions about how circuits involved in this form of motivation aligns with those driving motivated behavior at the human level. Further, the intrinsic self‐driven motivational deficit prevalent in apathy appears irrespective of homeostatic needs. Food or water‐motivated tasks have greater sensitivity to changes in appetite or metabolism, which may be impacted non‐specifically by pharmacological treatment or disease models, making behavioral interpretation difficult. Training the rodents to respond consistently and at a high enough level to observe drug or phenotype‐mediated changes can take weeks to months. This prolonged period of learning does not translate effectively to human equivalent tasks, which are achieved usually within a single session. Extended training times also bring higher associated costs (both in terms of time and expense). Related to this, the high cost of operant behavioral equipment, associated software and necessary space is a barrier to use in some laboratories.

The recently developed effort‐based forage (EBF) task is based on the intrinsic, species‐specific drive to forage for nesting material. In this task, the mouse is placed in an arena with access to both food and water and can freely choose to traverse a tube to a forage area. Here, the mouse can choose to forage nesting material by pulling it through apertures in a custom designed box. The act of foraging requires effort, which can be modulated by changing the aperture size. The amount of nesting material foraged and shuttled back to the main arena provides a readout of motivational state. The task has a broad range of applications across the fields of pharmacology and neuroscience. It has been used to test the acute or chronic effects of compounds on motivated behavior (Xeni et al., 2024, 2025) and can be utilized in model phenotyping studies to assess changes in effort‐based modulation of behavior and affective reactivity. As the task is driven by innate behavior, food/water restriction is not required to induce robust behavioral responses. Capturing behavior in the absence of an external physiological motivator such a food/water restriction allows for intrinsic motivation to be measured and for greater access to individual variability in motivated state. Of note, the absence of food and water restriction provides an additional rodent welfare benefit in line with the principles of refinement set out by the 3Rs (i.e., replacement, reduction, refinement). The task has further practical and translational benefits; in contrast to conditioning tasks, it does not require extensive training periods, providing researchers with a rapid behavioral readout ideal for time sensitive studies and more closely aligned with human learning rates. The task is built from open‐source components and dimensions, allowing labs to replicate the designs within their own institutions.

The EBF task has been used to test the effects of a range of clinically used compounds, including modulators of the dopaminergic and serotonergic systems (Xeni et al., 2024, 2025). With greater access to individual variability in motivational state, the task showed dose‐dependent differences in response to dopaminergic drugs dependent on intrinsic motivation level in healthy mice. The task has also been used to reveal important differences in antidepressant effect dependent on motivation task used, revealing that the task assesses a different form of motivation to that typically assessed in operant conditioning paradigms. The task has successfully been used to detect deficits in phenotypic models where motivational change is a known symptom, including healthy ageing and chronic corticosterone treatment (Xeni et al., 2024).

The standard EBF task set up facilitates the screening of acute pharmacological treatment. Across multiple test sessions equal to the number of doses tested, mice are required to forage under a moderate effort contingency over a 2‐hr period. Aspects of the task environment can be changed to test multiple behavioral dimensions that may impact on motivated behavior. This is particularly useful in the context of apathy syndrome, which consists of multiple dimensions of disrupted processing, including emotional/affective blunting in addition to deficits in motivation for reward (Ang et al., 2017; Marin, 1991). In phenotypic or chronic treatment studies, mice are taken through these versions of the task to build a comprehensive overview of factors contributing to motivational deficit.

Effort‐based modulation of behavior can be assessed by changing the effort required to forage from the nesting material box. Across three test sessions, mice are required to forage from the nesting material box containing apertures of varying sizes representing “easy”, “moderate”, and “difficult” levels. These varying effort contingencies are used to generate an effort curve, used to assess differences in effort‐based modulation of behavior between groups. Affective reactivity can be tested within the task by enlarging the forage area. Mice find larger spaces more aversive (Rex et al., 1998), and this principle can be applied to the foraging environment. Change in foraging behavior in response to a more aversive environment provides insight into affective reactivity. Mapping behavioral responses to these changing environmental contingencies can provide insight into whether a phenotype aligns with apathy or an alternative driver of motivational change (Xeni et al., 2024).

Overall, the EBF task provides a cost‐effective and comprehensive analysis of mouse motivational state independent of physiological state change induced by food/water restriction. It requires limited human‐mouse interaction, minimizing the risk of experimenter bias. It provides unique insight into intrinsic motivation, complementing findings from the effort for reward task. It can be utilized in pharmacological and phenotypic studies, with the potential to extend into the fields of circadian and stress research. In this article, we describe the methods used to habituate the mice to the EBF task and set up of the task arena. Basic Protocol describes the EBF task protocol used to test the impact of acute pharmacological manipulation on motivational state. Alternate Protocol 1 describes the effort curve paradigm used to test effort‐based behavioral modulation under varying effort contingencies. Alternate Protocol 2 describes the larger foraging area variation of the EBF task, used to test the affective reactivity of the mouse to an aversive environment. These Alternate Protocols can be used in the characterization of motivational deficit in a phenotypic model.

*NOTE*: All protocols involving animals must be reviewed and approved by the appropriate Animal Care and Use Committee and must follow regulations for the care and use of laboratory animals.

## ANIMAL HUSBANDRY AND ARENA SET UP

This Support Protocol provides important preparation guidance and the experimental set up neccessary to run the EBF task successfully.

### Materials

**Figure 1:**
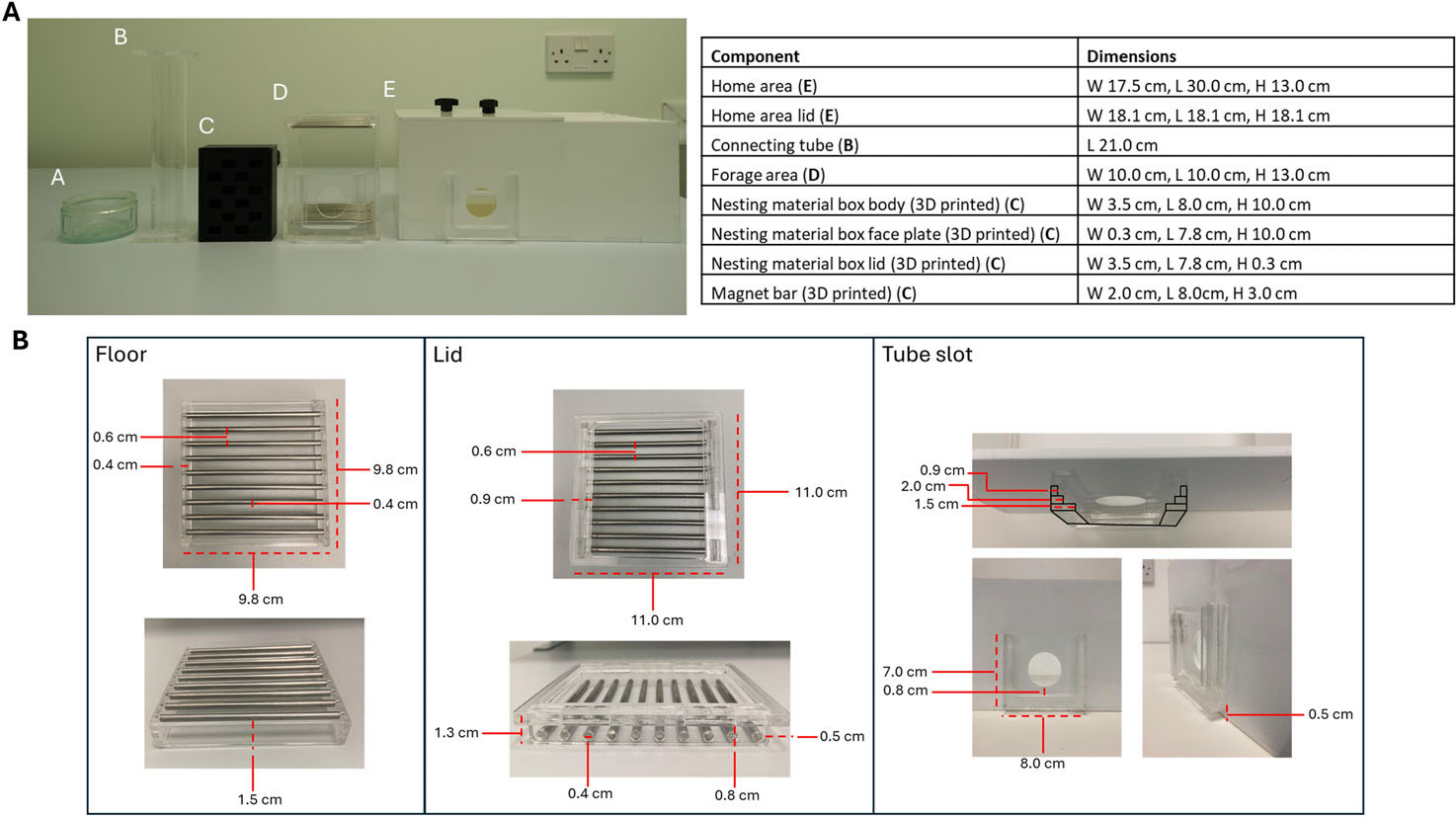
Dimensions of main and subcomponents of the EBF task arena. (**A**) Photograph depicting the main components of the EBF task arena with associated table of dimensions. (**B**) Dimensions for components of the forage area floor and lid and the tube slot. The home area, connecting tube and foraging area are built from Perspex. The nesting material box is printed using PLA filament. Bars are stainless steel with a diameter of 4 mm.

Mice [the task has been validated in C57bl/6JOlaHsd mice (Envigo) but other strains may be used]
Chosen drug(s) in solution if following the acute pharmacological testing protocol
Effort‐based forage arena(s); dimensions for building can be found in Figure 1
  We recommend the use of 4 to 6 arenas to facilitate parallel running of mice and multiple behavioral runs throughout the day.
Nesting material box with the 1.5‐cm^2^ and 1.0‐cm^2^ aperture faceplates (interchangeable)
Tweezers
Magnets to fit into the magnet bar and nesting material box (N35 neodymium magnet, 15‐mm diameter × 2‐mm thick, 1.8‐kg pull)
3D printed components, openly available from https://github.com/meganjackson13/Bedding‐box‐3D‐files
Sizzlenest (or similar) nesting material (Datesand)
Small cardboard tube for home cage enrichment and for use in taking the mouse in/out of the arena (LBS)
Bedding substrate (use the same substrate as used in the home cage)
Water bottle (use the same bottle used in the home cage)
Lab chow (use the same lab chow used in the home cage)
A small glass bowl for 4 × lab chow pellets
Weighing scales with accuracy to 0.01 g to weigh the nesting material box pre‐ and post‐ session
A rack, table, or other equivalent flat surface to place arenas on
Optional: A passive sensor system may be utilized to capture non‐specific movement in the foraging area
  Designs to build an inhouse monitoring system are openly available and low cost (Brown et al., 2016).

**Figure 2:**
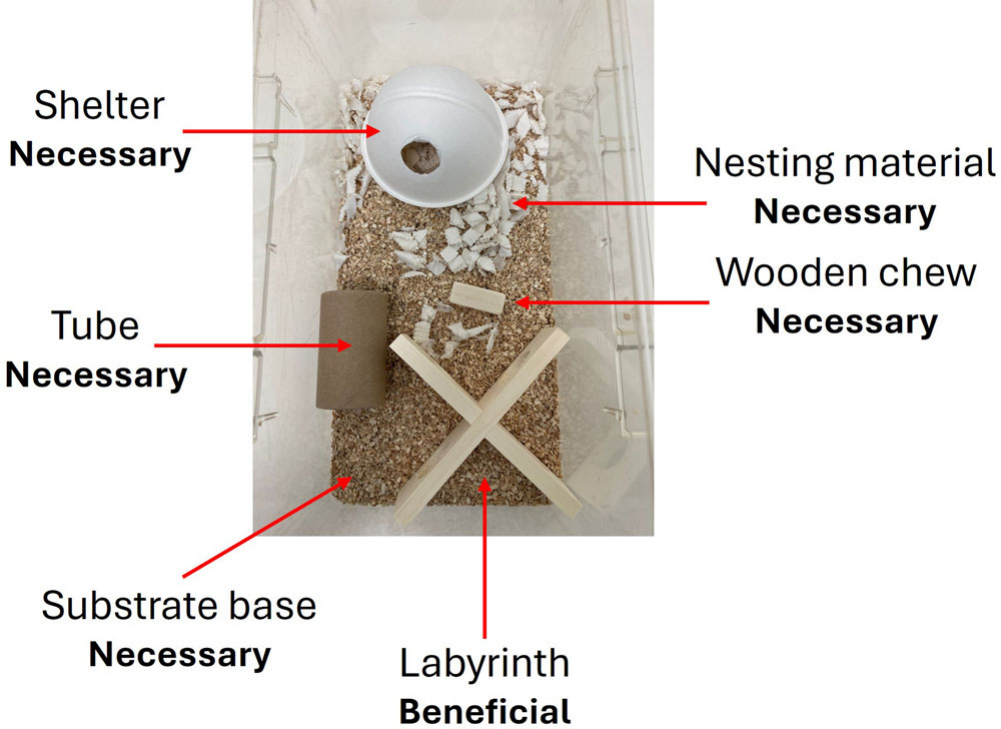
Suggested home cage enrichment.

1 Studies involving live animals require ethical approval before the study can begin. Ensure that you have obtained ethical approval from the appropriate body and follow institutional guidelines for laboratory animal care.
2 Allow mice to acclimatize for at least one week to a new unit/environment before onset of studies. Stress induced by transportation can induce physiological and/or behavioral changes that may affect task output (Tuli et al., 1995).
3 House mice in suitable caging.
  As a guide, we use open top Techniplast 1284 caging to house 1 to 2 mice per cage. We recommend singly housing male mice to reduce psychosocial stress and risk of injury associated with fighting in grouped housing. There is evidence to suggest that sexually mature male do not naturally live in groups (Salamone et al., 2018) and may be in a more positive affective state when housed alone (Davies et al., 2025). Note this does not necessarily apply to female mice.
4 Animal holding rooms must be kept at a consistent temperature (21.5° ± 1°C) and humidity (55 ± 10%). Animals must be tested in their active (dark) phase. We recommend a 12 hr‐12 hr light‐dark cycle, where lights turn off in the daytime (e.g., 8:15 a.m.). Dim red lighting may be used to allow the experimenter to operate in perceived darkness.
5 Where possible provide mice with home cage enrichment (suggested setup provided below, Fig. 2). Ensure mice are provided with a tube that can later be used to remove mice from the experimental arena.

6 Food and water may be provided ad libitum throughout the study. Palatable food reward may be used to facilitate handling habituation (see below).
7 Before task onset mice must be habituated to handling by the experimenter to reduce associated stress as described by the 3Hs Initiative: Housing, Handling and Habituation. A handling protocol is available on the 3Hs Initiative website (see Internet Resources). Handling habituation is complete when mice show limited avoidance to being cupped in the home cage and show limited attempts to escape from the open palm.
  Note that mice are unlikely to perform effectively if they are stressed by human interaction.

#### Daily preparation of experimental setup

**Figure 3:**
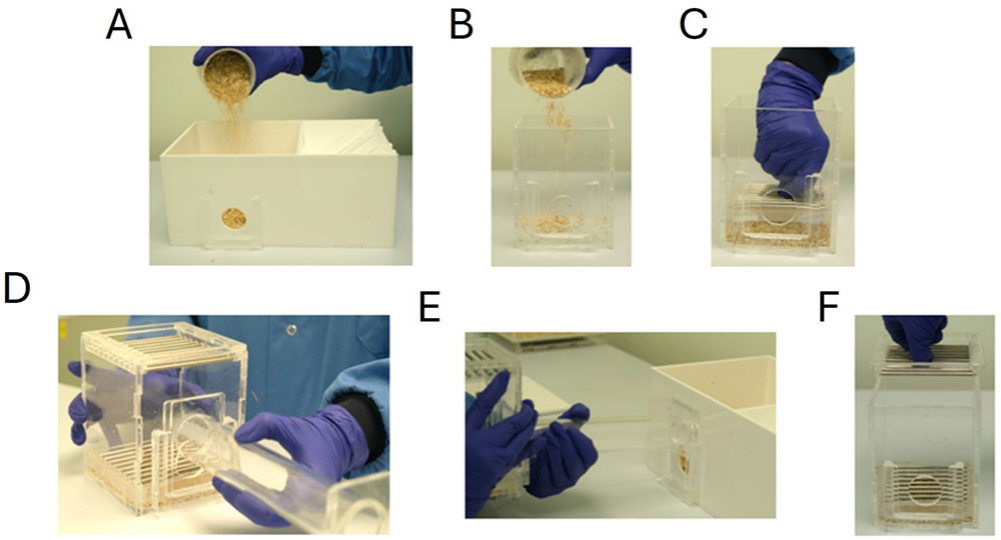
Set up of the EBF task. Photographs illustrate: (**A**) pouring a layer of woodchip into the home box; (**B**) pouring a layer of woodchip into the foraging box; (**C**) putting the barred floor into the foraging box; (**D**) attaching the tube to the foraging box; (**E**) attaching the tube to the home box; and (**F**) placing the barred ceiling on the foraging area.

8 Testing of compounds, phenotypes or environmental manipulations must be counterbalanced to account for the effects of time or order of running. Ensure you bring this information with you on test days.
9 Set up the arenas as described in Figure 3 and the steps below. The use of multiple arenas (4 to 6) is advised, allowing mice to run in parallel and for multiple behavioral runs across the day.

**Figure 4:**
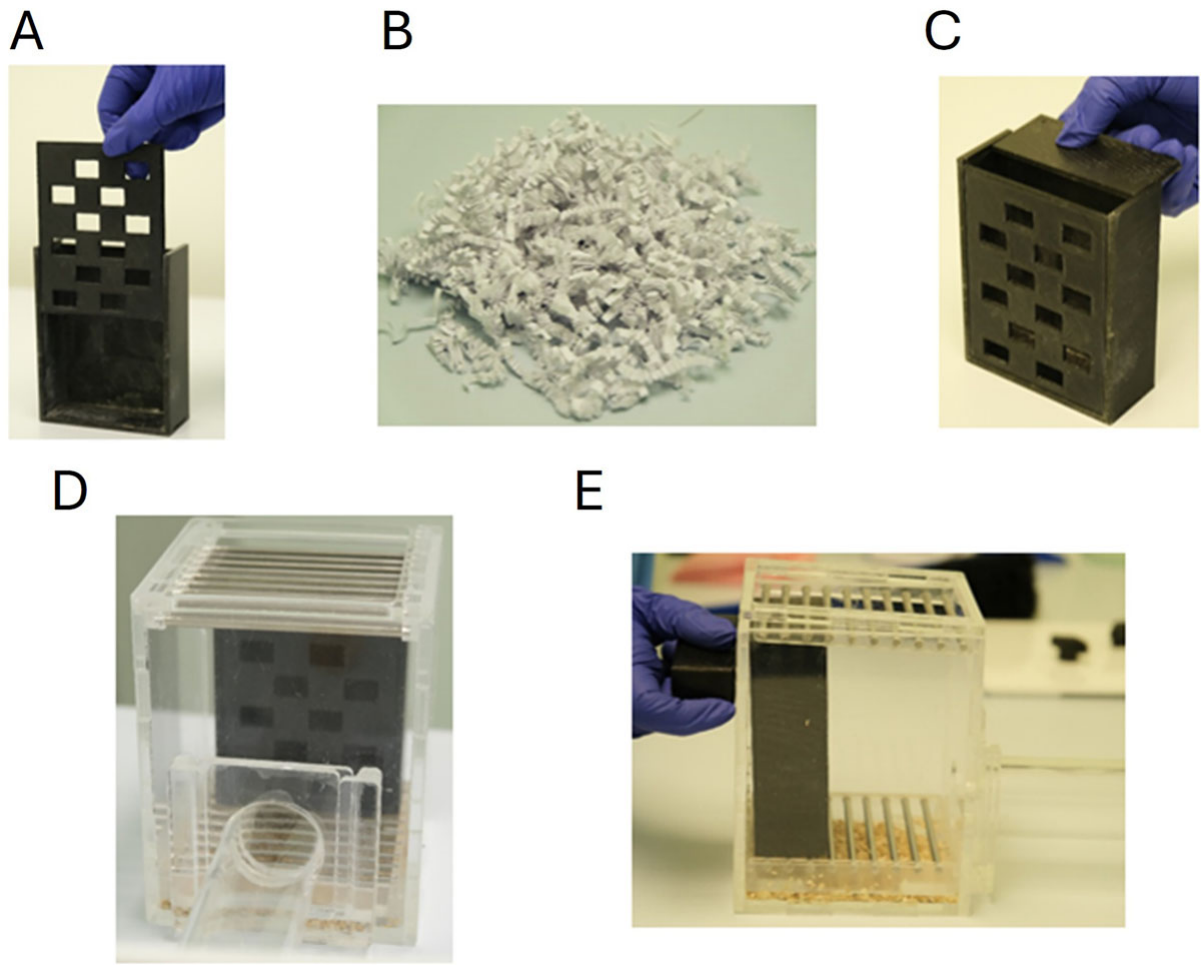
Set up of the nesting material box inside the arena. Photographs illustrate: (**A**) the easy aperture faceplate sliding into the nesting material box; (**B**) Sizzlenest material; (**C**) the lid of the nesting material box sliding on; (**D**) nesting material box placed in the foraging area with apertures facing the tube; and (**E**) securing the nesting material box with a magnet. Note that for the purpose of this figure, images show placement of the nesting material box and do not contain any Sizzlenest.

10 Pour a thin layer of woodchip (or whichever substrate is used as bedding in the home cage) into the home box (Fig. 3A). This equates to ∼70 g of woodchip but this may differ depending on substrate. Shake the home area to ensure that woodchip is evenly distributed across the floor.
11 Pour a thin layer (∼10 g) of woodchip into the foraging box (Fig. 3B). Shake the box to ensure woodchip covers the whole floor of the box.
12 Place the barred floor into the foraging box so the bars are horizontal to the hole in the box, pushing down so it sits directly on top of the woodchip (Fig. 3C). If you have difficulty pushing the barred floor on top of the woodchip, the layer of woodchip may be too thick.
13 Fit the tube onto both boxes by sliding the tube into the tube holder on each box (Fig. 3D and 3E) making sure it is secured to both boxes.
14 Place the barred lid on the foraging box again horizontal to the tube (Fig. 3F).
15 If progressing past the initial arena habituation steps (described below in Basic Protocol), the nesting material box must also be set up according to the following steps and outlined in Figure 3.
16 Slide the relevant aperture faceplate into place in a nesting material box (Fig. 4A).

**Figure 5:**
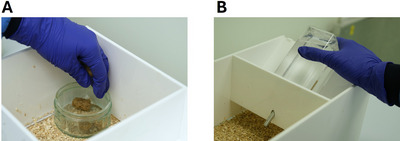
Placement of food and water within the task arena. Photographs illustrate: (**A**) standard lab chow pellets in a bowl in the home area; and (**B**) water bottle placed in the holder on the home area.

17 Fill this box with 18 g of white Sizzlenest (Fig. 4B). Record the weight of the box holding 18 g of Sizzlenest (we recommend taking the lid off to weigh the box).
  Take care to ensure that the Sizzlenest is evenly spread through the nesting material box and not more compact in some areas than others. If the Sizzlenest does not fill the box to the top, this indicates that the density of nesting material pushed in is too high.
18 Slide the lid onto the box (Fig. 4C), use tweezers to tuck any loose strands in.
19 Use tweezers to pull a small amount of Sizzlenest through all the holes of the faceplate, only enough so that the material can be reached by the mouse.
20 Place the nesting material box onto the back wall of the foraging box (Fig. 4D), and secure using the magnet on the outside of the foraging box (Fig. 4E). Attach the barred roof.
21 Place 4 standard lab chow pellets into a small bowl and place this in the back left corner of the home box (Fig. 5A).

22 Place a water bottle in the holder on the home box (Fig. 5B).
23 Record the ID of the mouse, its assigned arena, and the weight of each nesting material box preceding the start of the task.

#### Pharmacological considerations

In the case of acute pharmacological studies, carefully consider the dose range used. Ensure that your chosen doses do not induce non‐specific motor effects, which may make behavioral interpretation difficult. You should also carefully consider route of administration. Where possible, try to use a method that requires the least amount of restraint. For example, the use of voluntary oral dosing in a palatable solution as described by the 3Hs Initiative (see Internet Resources).

## HABITUATION AND ACUTE PHARMACOLOGICAL MANIPULATION

This protocol describes the use of the EBF task to test the impact of acute pharmacological manipulation on motivation to forage. During habituation, animals become familiar with the task environment. Once habituated, mice will reliably forage nesting material from the nesting material box and shuttle most of it back to the home area. Motivated behavior can be quantified by measuring weight of material foraged and shuttled. This assay allows the addition of pharmacological manipulation preceding a forage session to quantify the effect of different drugs (or indeed other acute manipulations) on motivated behavior.

### Materials

See Support Protocol

#### Habituation

**Figure 6:**
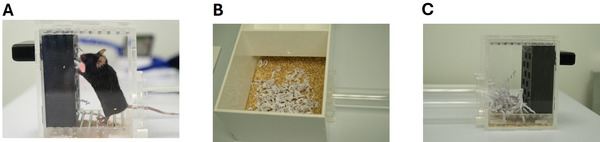
Photographs illustrate: (**A**) a mouse foraging from the nesting material box; (**B**) nesting material shuttled to the home area of the forage arena; and (**C**) nesting material left in the foraging area on top of the barred floor after a test session.

1 For the first stage of habituation, set up the arenas as described in Support Protocol, steps 9 to 14.
2 Place the mouse into the home box via a tube or cup handling. Close the lid of the home box. Let the mouse explore the arena for 10 min. You may leave the room during this period or choose to score bouts of entry into the forage area as a measure of exploration during habituation.
3 Take the mouse out of the arena by placing the cardboard tube at the entrance to the tube and wait for the mouse to enter. Remove the cardboard tube with the mouse in and return the mouse to its home cage.
  It may take a while for the mouse to enter the tube but after it has done this will become more efficient than cup handling.
4 Repeat step 2 and 3 for the following 2 days but for 5 min each day.
5 For the fourth day of habituation, set up the arena following Support Protocol, steps 9 to 23. Use the easy 1.5‐cm^2^ aperture faceplate when setting up the nesting material box.
6 Place the mice into the home boxes by cup handling or using a cardboard tube and put the lid on. Leave the mice for 4 hr. The mouse should approach the nesting material box and spontaneously forage throughout the session (Fig. 6A, Video 1).

**Video 1:** Example of a male C57bl/6 mouse foraging from the nesting material box. Note that the mouse disengages from the nesting material box throughout to explore the forage area, this is expected and part of a normal behavioral repertoire.

**Figure 7:**
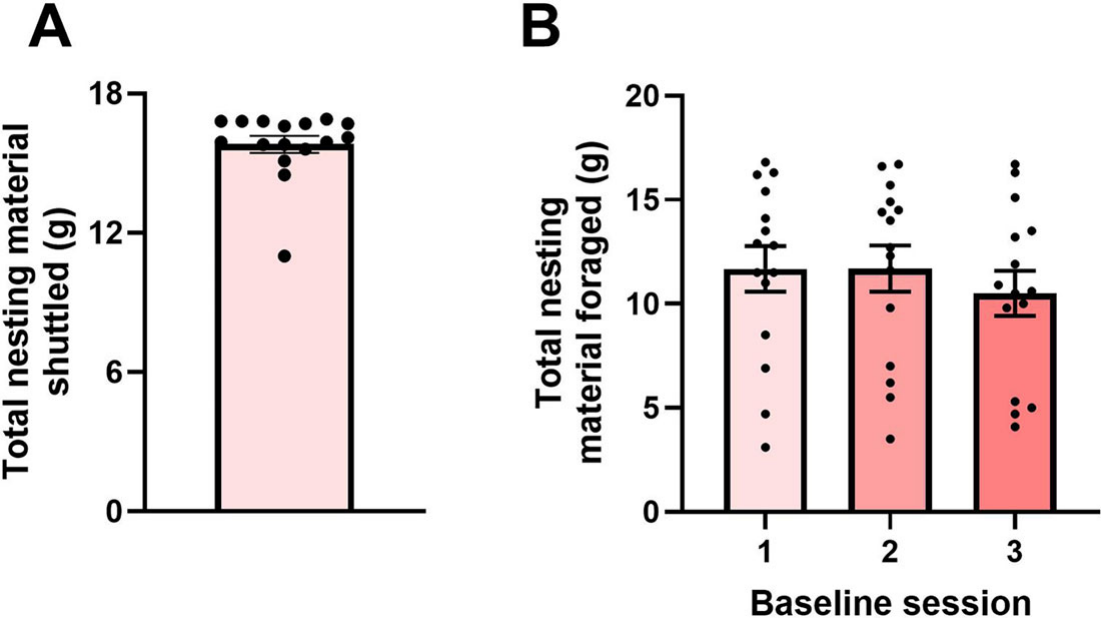
Habituation and baseline data. (**A**) Total nesting material shuttled in a 4‐hr habituation session using the easiest (1.5‐cm^2^) apertures. Most mice should forage at the upper limit and may create an observable “ceiling effect”. Some mice may forage at a lower level as shown. In this instance, the habituation session may be repeated to ensure that this lower foraging level is not stress mediated. (**B**) Three 2‐hr baseline sessions using the moderate (1.0‐cm^2^) apertures. These conditions induce variation in foraging performance in mice, and you should expect to see a widespread range in foraging as shown. The expected mean cohort performance for a male C57bl/6jOlahsd mouse from Envigo is ∼10 g. There should be no statistical cohort‐level trend in foraging performance across three sessions before starting pharmacological studies.

7 Remove the mice from the home boxes as described in step 3.
8 Remove the barred ceiling of the foraging area and the magnet. Weigh the nesting material box and record its new weight. This will provide a measure of how much nesting material has been foraged from the box. Most of the foraged material should have been shuttled to the home area (Fig. 6B).
9 Pick out any nesting material left on top of the barred floor of the foraging area (Fig. 6C), weigh this and record. This facilitates calculation of total nesting material shuttled (see the Understanding Results section).
  Only pick out nesting material in the foraging area that could be reached by the mouse, i.e., not material under the barred floor.
10 Repeat these steps for all mice in the cohort.
  Mice should shuttle most of the foraged Sizzlenest back to the home box, if this is not the case, consult the Troubleshooting section for further information and repeat 4 hr habituation session.
11 Habituation is complete when most of the mice forage and shuttle most of the nesting material (>12 g total shuttled, >80% nesting material shuttled). See Figure 7A for expected habituation results.

#### Baseline

12 Set up the arena following Support Protocol, steps 9 to 23.
13 Run the experiment for 3 sessions of 2 hr, using a moderate aperture faceplate (1.0 cm^2^) in the nesting material box.
14 If the results show a stable rate of foraging (i.e., no statistical increase or decrease in foraging or shuttling level over sessions at the cohort level), you may proceed to pharmacological testing (see Fig. 7B).

#### Acute pharmacological testing

15 Allocate drug conditions randomly to A, B, C and D so that the experimenter is blind to treatment.
16 Counterbalance order of treatment using a Latin square design as shown in Table 1.

**Table 1:** A 4 × 4 Counterbalanced Design for a Pharmacological Study*^a^*

4 × 4	Vehicle and 3 doses			
Animal ID	Session 1	Session 2	Session 3	Session 4
Mouse 1	A	B	C	D
Mouse 2	C	A	D	B
Mouse 3	B	C	A	C
Mouse 4	D	C	B	A

^*a*^ Each letter corresponds to a blinded drug dose. In this within‐subject design, each mouse receives each dose of drug across 4 test sessions. This design can be scaled up to the number of mice within the cohort by e.g., repeating the proposed pattern.

17 Administer the drug to the mice using the most appropriate and refined dosing method, at a predetermined pre‐treatment time appropriate to the drug pharmacokinetics.
18 Run the task for 2 hr using the moderate aperture faceplate (1.0 cm^2^) inside the nesting material box.
19 Repeat steps 17 and 18 until each mouse has received each dose of the drug.

**Figure 8:**
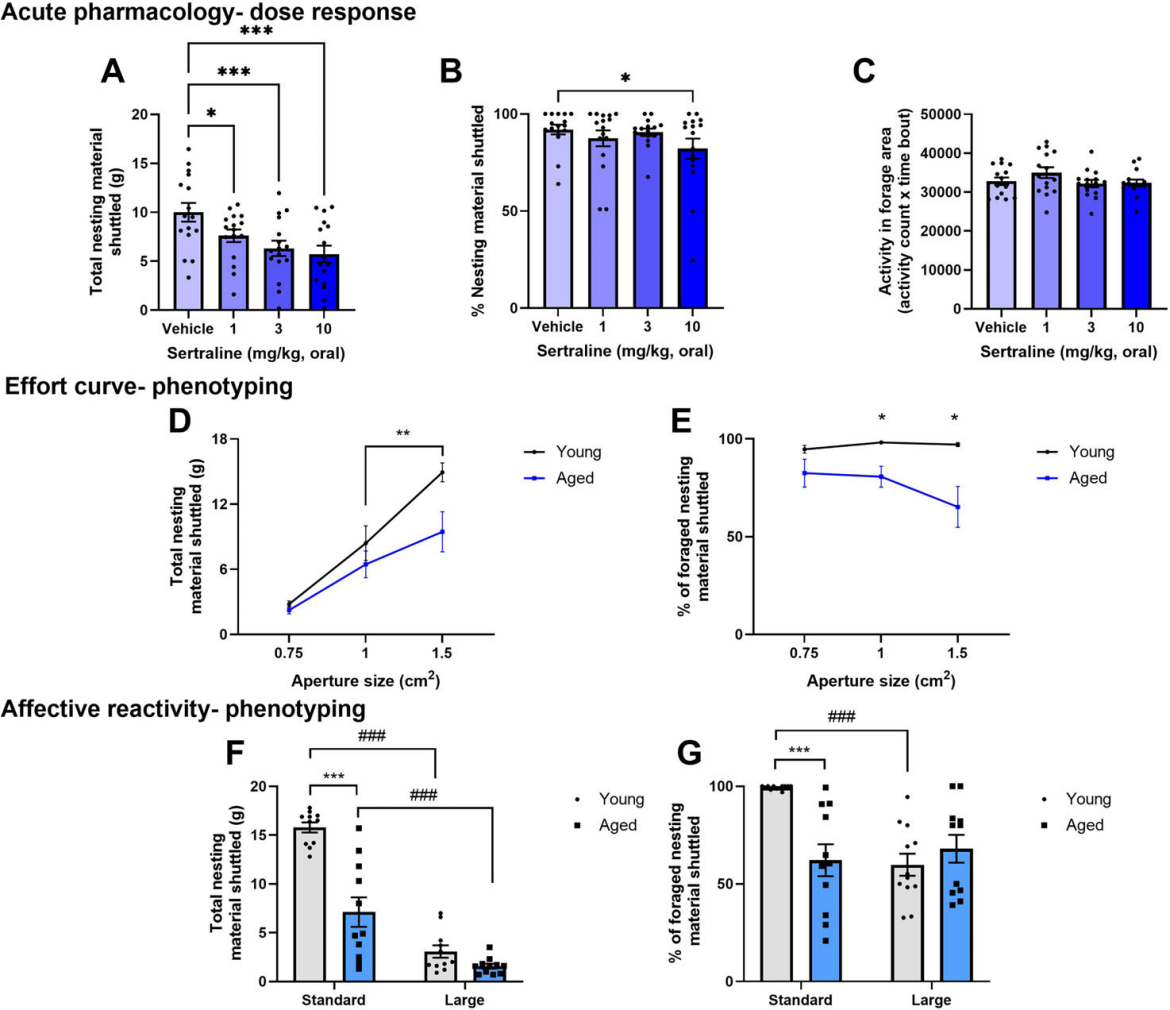
Example results from Basic Protocol and Alternate Protocols 1 and 2. (**A**) A dose‐response to sertraline in total nesting material shuttled, where sertraline reduces total nesting material shuttled at all doses. (**B**) Sertraline reduced % nesting material shuttled at the highest dose. (**C**) Sertraline did not alter home cage activity. Statistical differences are depicted using * *p* < .05, **p < .01, ***p < .001, within‐subject analysis. Data are presented as mean ± *SEM* with individual data points overlaid. (**D**) The effect of ageing on effort curve performance. Nesting material shuttled is reduced at the 1‐cm^2^ aperture compared to the 1.5‐cm^2^ aperture in young mice as expected, however this is not the case in aged mice. (**E**) Aged animals show a reduction in % nesting material shuttled at the 1‐ and 1.5‐cm^2^ apertures compared to young mice. Statistical differences are depicted using * *p* < .05, **p < .01, between‐subject analysis. Data are presented as mean ± *SEM*. (**F**) A reduction in nesting material foraged in the larger forage area vs the standard forage area in both young and aged animals. (**G**) % nesting material shuttled is reduced in a larger forage area in young but not aged mice. ***p < .001, between‐subject comparison, ^###^ *p* < .001, within‐subject comparison. Data are presented as mean ± *SEM*. Figures altered from Xeni et al. (2024, 2025).

**Figure 9:**
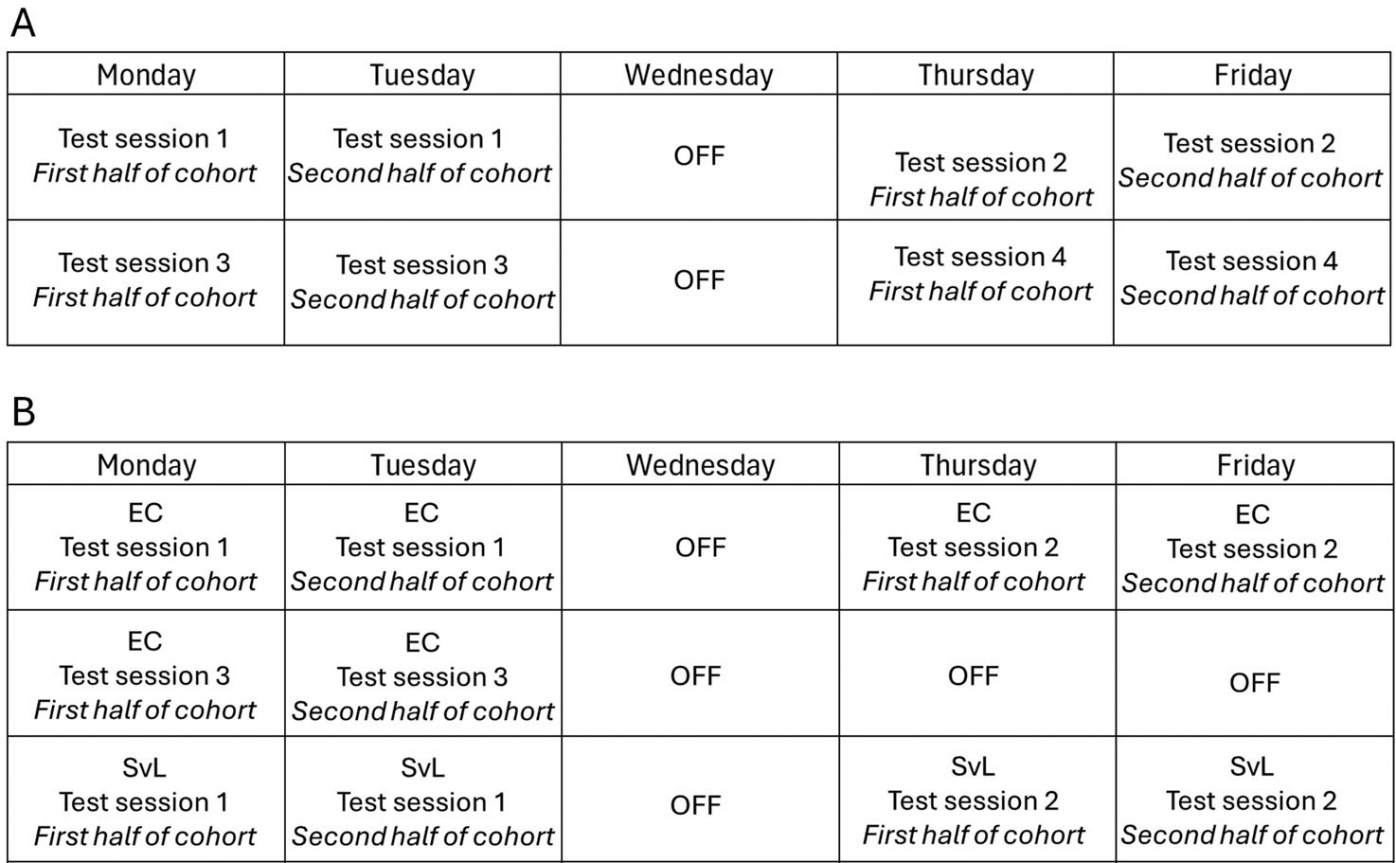
Proposed timetables for experimental planning. (**A**) A proposed timetable for a within‐subject dose response study with vehicle and 3 doses. Each test session is split over two days to facilitate multiple behavioral runs. Each test session is separated by 2 days to allow for drug washout. If the number of behavioral runs are fewer, testing can be confined to 1 day per session. (**B**) A proposed timetable for completing the effort curve and standard vs large paradigms. Each test session is split over 2 days to facilitate multiple behavioral runs. Each test session is separated by 2 days to align with drug studies and prevent immediate experience from impacting on performance. If number of behavioral runs are smaller, testing can be confined to one day per session. EC‐effort curve, SvL ‐ standard versus large.

For example, a study using a vehicle group and 3 different doses of drug requires 4 test sessions. Ensure each test session is separated by at least 2 days to ensure sufficient washout of the drug between test days. A suggested timetable is provided in Figure 9. Allow at least 2 days between different drug studies. See Figure 8A for example dose response data.

## Alternate Protocol 1 THE EFFORT CURVE PARADIGM

This protocol introduces changes to the effort required to forage nesting material by using different faceplate aperture sizes. This creates a measure of the ability of the mouse to adapt their foraging behavior to different effort contingencies, which can be used to identify any changes in effort‐modulated behavior.

### Additional Materials (also see Support Protocol)

Nesting material box with the 1.5‐cm^2^, 1.0‐cm^2^ and 0.75‐cm^2^ aperture faceplates (interchangeable)

**Figure 10:**
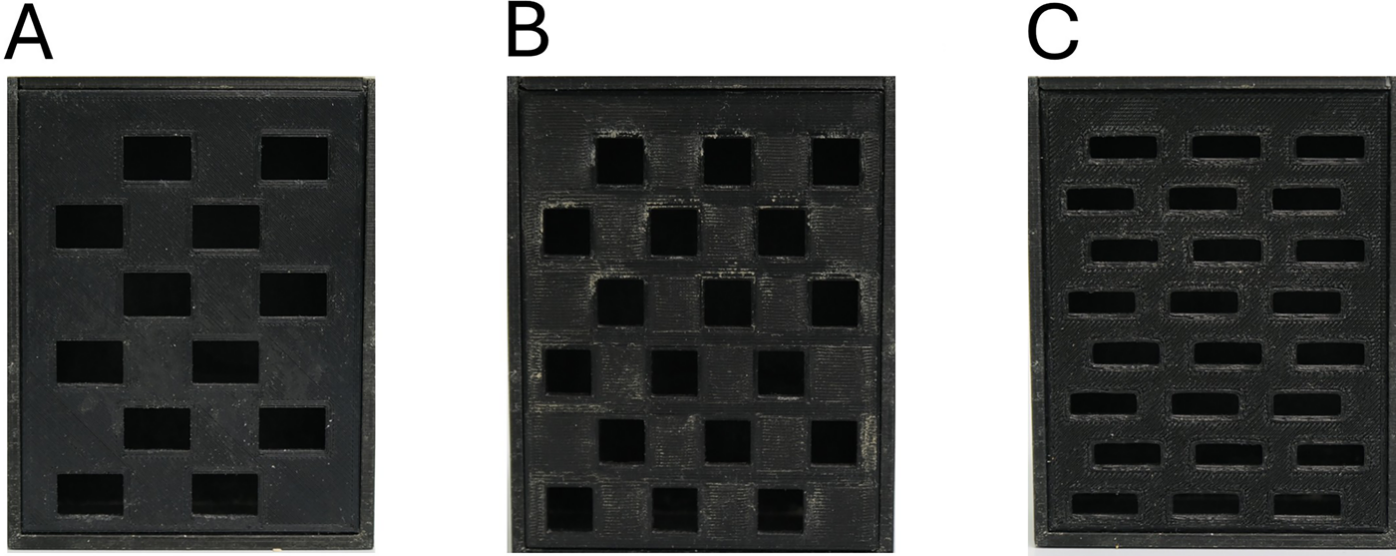
Photographs illustrate (**A**) easy 1.5‐cm^2^ aperture faceplate, (**B**) moderate 1.0‐cm^2^ aperture faceplate, and (**C**) difficult 0.75‐cm^2^ aperture faceplate.

1 Generate your desired disease/chronic treatment model.
2 Set up the arenas and habituate the mice as previously described in Support Protocol, steps 9 to 23.
3 Before starting the first test session, allocate mice to one of three faceplates used for the nesting box [easy, 1.5 cm^2^ (Fig. 10A), moderate, 1.0 cm^2^ (Fig. 10B), and difficult, 0.75 cm^2^ (Fig. 10C)] per session according to a counterbalanced design. An example is provided in Table 2.

**Table 2:** A 3 × 3 Counterbalanced Design for the Effort Curve Paradigm*^a^*

3 × 3	3× effort contingencies		
Animal ID	Session 1	Session 2	Session 3
Mouse 1	Easy	Moderate	Difficult
Mouse 2	Difficult	Easy	Moderate
Mouse 3	Moderate	Difficult	Easy

^*a*^ In this within‐subject design, each mouse receives each aperture size across 3 test sessions. This design can be scaled up to the number of mice within the cohort by e.g., repeating the proposed pattern.

4 Record nesting box weight change and nesting material left on top of the barred floor as previously described in Basic Protocol, steps 8 and 9.
5 Repeat the experiment for three sessions in total, so that all mice undergo a test session with each aperture faceplate. Ensure at least a 2‐day gap between test sessions so that previous foraging experience from the preceding test session does not impact on test performance in the following session.

## Alternate Protocol 2 AFFECTIVE REACTIVITY TEST

This protocol introduces changes to the foraging environment by increasing the size of the foraging area. This protocol is designed to measure affective reactivity to a more open/averse environment by comparing foraging and shuttling behavior in a standard vs larger foraging area.

### Additional Materials (also see Support Protocol)

Nesting material box with the 1.5‐cm^2^ aperture faceplate
A larger version of the forage area component of the arena (width 21.0 cm, length 21.0 cm, height 14.0 cm)

**Figure 11:**
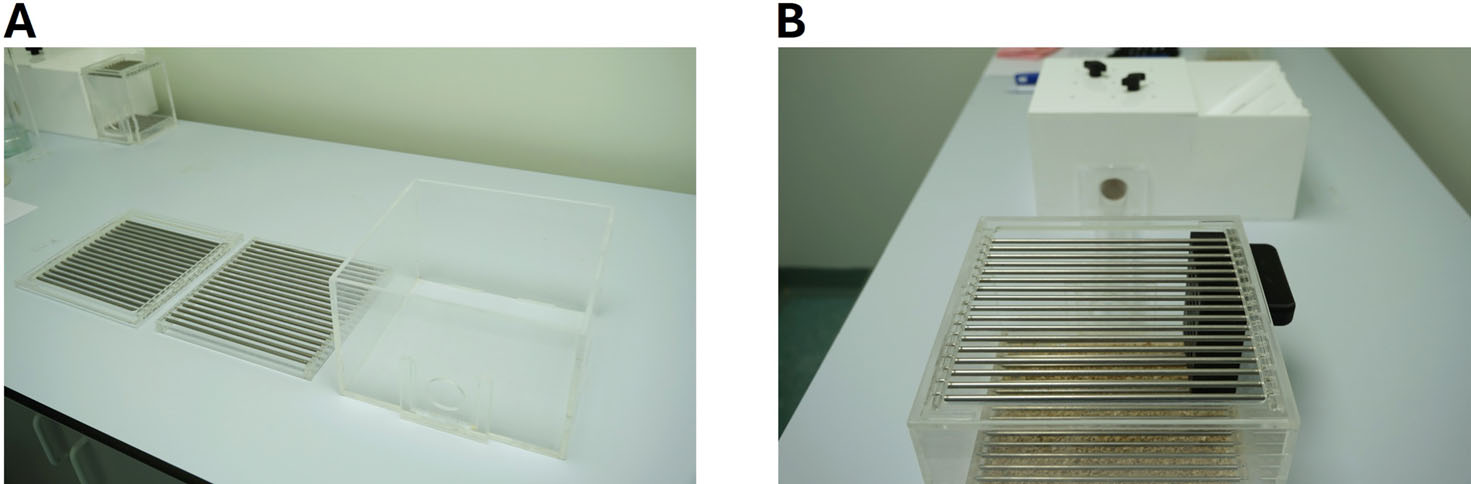
Photographs illustrate (**A**) the larger sized foraging area, and (**B**) the placement of the nesting material box within the larger arena.

1 Set up the arenas as previously described in Support Protocol, steps 8 to 23. The nesting box with the 1.5‐cm^2^ apertures should be used throughout, but the size of the foraging area will change across sessions.
2 Use ∼80 g woodchip to cover the floor of the larger foraging area. The nesting material box should be placed on top of the barred floor, on the left‐side wall (from the perspective of the mouse entering the forage area from the tube) as close to the tube as possible.
3 Before the first test session, allocate half the mice to the standard (Fig. 11A) or large (Fig. 11B) foraging area (see example in Table 3).

**Table 3:** A 2 × 2 Counterbalanced Design for Affective Reactivity Testing*^a^*

2 × 2	2× forage area sizes	
Animal ID	Session 1	Session 2
Mouse 1	Standard	Large
Mouse 2	Large	Standard

^*a*^ In this within‐subject design, each mouse receives each forage area size across two test sessions. This design can be scaled up to the number of mice within the cohort by e.g., repeating the proposed pattern.

4 Record nesting box weight change and nesting material left on top of the barred floor as previously described in Basic Protocol, steps 8 and 9.
5 Repeat this for two sessions total so that all mice undergo both conditions. Ensure at least a 2‐day gap between test sessions so that foraging experience from the preceding test session does not impact on test performance in the following session.

## COMMENTARY

### Critical Parameters

#### Mouse habituation to experimenter

As described above, it is crucial that mice are habituated to either tube or cup handling before task onset. The task is highly sensitive to stress, which may be induced by handling without habituation. Stress can suppress foraging behavior or increase variation in foraging behavior leading to longer task habituation times. Do not tail handle mice.

#### Task environment (external)

Ensure that the task environment is quiet with limited activity in the room. Lights should be switched off or a dim red light used if necessary. Consider where the arenas will be placed within a room and ensure that a flat surface is used. Minimize external noise as much as possible. There is evidence to suggest that height and proximity to the door can change normal behavior in mice (Ueno et al., 2023). Ideally, arenas should not be positioned facing the door. Use of moveable barriers may be used to block view of the door. Ensure that treatment or intervention groups are counterbalanced by arena position to ensure effects are not driven by non‐specific environmental factors. We suggest that in mixed‐sex studies female mice and male mice do not share boxes between behavioral runs to reduce change of distraction induced by scent not fully removed by cleaning.

#### Task environment (internal)

Ensure that the substrate (e.g., woodchip, sawdust) used at the base of the arena is the same as that of the home cage to reduce stress associated with novelty. Carefully consider which nesting material is selected for the task. Sizzlenest proved optimal in terms of rate of foraging within the task and we suggest its use where possible. Mice show strong motivation to forage this material, potentially due to its suitability for building a structured nest not achievable with softer bedding (Bárdos et al., 2022). Nesting material should be packed into the nesting material box in a consistent manner across test sessions. Differing densities of packed nesting material between sessions may induce variability. To further mitigate this, ensure the same experimenter is packing the nesting material across the study to improve consistency.

### Troubleshooting

See Table 4 for common problems and troubleshooting solutions for the EBF task.

**Table 4:** Troubleshooting Guide for the EBF Task

Problem	Possible cause	Solution
Low foraging levels during habituation session in healthy mice	Lighting levels are too bright	Ensure that the task is run in complete darkness or dim red lighting; check your red bulbs are not emitting white light as red lamps can degrade over time
	Mouse finds arena aversive	Add additional arena habituation sessions to reduce anxiety associated with the arena; if an additional 3 sessions is not sufficient then lack of foraging is likely driven by another cause
	Mouse is not habituated to handler	Ensure that your mice are habituated to handling and the experimenter as per the 3Hs guidelines
	Selected nesting material is not suitable	Different nesting materials may have different packing densities or reward value that may alter the foraging behavior of the mice; start with packing your chosen material less densely to ensure mice are motivated to forage material; if mice continue to not forage, try swapping to a different nesting material
Low levels of shuttling behavior during habituation in healthy mice	As above	As above
High variation in foraging levels within mouse	Material is packed into the nesting material box at different densities across sessions	Ensure that nesting material is filled in a consistent manner as inconsistencies can change effort required to forage; mitigate this by using the same or smaller number of experimenters within‐experiment
Failed effort curve	Material is packed into the nesting material box at different densities across sessions	Ensure that nesting material is filled in a consistent manner as inconsistencies can change effort required to forage; mitigate this by using the same or smaller number of experimenters within‐experiment

### Statistical Analysis

Statistical analysis is dependent on choice of study design and distribution of the data. The Shapiro‐Wilk test is appropriate for assessing normality in the sample size typically used in this type of work (range of *n* = 8 to 24 per group). In the case of an acute within‐subject pharmacological dose response study, groups should be compared with a repeated measures one‐way ANOVA (or non‐parametric equivalent) and appropriate post‐hoc tests if main effects are observed. If taking a model or intervention through the different versions of the task (Alternate Protocol 1, the effort curve; Alternate Protocol 2, the standard vs large forage area paradigms) then a two‐factor analysis may be employed where interactions between the model and environmental contingencies may be analyzed e.g., effort level*model, arena size*model. The significance threshold is defined as *p* < .05. Outliers are defined as those 2 standard deviations away from the group mean. Outliers may be removed from the data set or replaced with the group mean to conserve repeated measures analysis. Experimenters should make themselves aware of the limitations associated with each method.

### Understanding Results

The task produces three main outputs with a potential fourth output if passive infrared sensors are integrated into the task set up. The outputs are as follows.

(1) Total nesting material foraged (g). This value represents the total amount of nesting material pulled from the nesting material box, irrespective of whether the nesting material is shuttled. This value is obtained by weighing the nesting material before and after the session and is calculated by *weight of nesting material box pre‐session – weight of nesting material box post session*. The resulting value is the amount of nesting material foraged in g.
(2) Total nesting material shuttled (g). This value represents the total amount of nesting material pulled from the nesting material box and then carried (shuttled) to the main home area. This value is obtained by weighing the nesting material before and after the session in addition to weighing the amount of nesting material left on top of the barred floor in the open forage area. Total nesting material shuttled is then calculated by *total nesting material foraged – amount of nesting material left in open*. The resulting value is the total nesting material shuttled to the main home arena.
(3) % of foraged nesting material shuttled. This value represents the amount of nesting material shuttled as a percentage of the total amount of nesting material foraged. It is calculated by (*total nesting material shuttled/total nesting material foraged)* × *100*. The resulting value provides a readout of amount of nesting material shuttled normalized to overall foraging level, expressed as a percentage.
(4) Total activity (if passive infrared sensors are used). This value represents the amount of general movement in the forage area, read by passive infrared sensors placed above the forage area. The sensors output an activity level value every 10 s. The value represents the % of movement within that 10 s time bin. Total activity within a given time range (e.g., duration of the task) can be calculated by using area under the curve (AUC) analysis.

#### General interpretation

While total nesting material foraged (g) provides a general indication of engaging with foraging behavior, total nesting material shuttled (g) provides an indication of a more goal‐directed behavior. A reduction in these outputs is indicative of a lower motivational state. It is important to be aware that other behavioral and external factors may impact on foraging activity and should be controlled for where possible using behavioral control tests. While related to these two output measures, % nesting material shuttled gives an additional indication of normalized proportion of foraged nesting material shuttled to the home area. A reduction in this measure indicates that the mouse is shuttling a lower proportion of foraged nesting material. Use of the affective reactivity version of the task indicates that this measure appears sensitive to modulation of affective reactivity in phenotypic models (Xeni et al., 2024). Leaving greater levels of nesting material in the open may indicate greater levels of anxiety‐related behavior. Total activity provides an indication of non‐specific movement in the foraging area. No change in this measure suggests overall movement has not been impacted by treatment/phenotype, providing a behavioral control specific to the task environment.

#### Data plotting

##### Dose response output measures

A bar chart with individual data points and an indication of statistical variation is appropriate (see Fig. 8). For normally distributed data, the mean and *SEM* or *SD* may be used. For non‐parametric data, the median and interquartile range may be used. Consider using different colors or symbol shapes to help visually differentiate between doses.

##### Effort curve output measures

An X–Y graph with an indication of statistical variation is appropriate (see Fig. 8). Where multiple groups have been tested, consider using different colored lines to help visually distinguish groups.

##### Affective reactivity (standard vs large forage area) output measures

Where two different groups are compared, a grouped bar chart with individual data points and indication of statistical variation is appropriate (see Fig. 8). Where only one group is used, a bar chart is appropriate.

### Time Considerations

Allow a minimum of 4 days for task habituation, with a leeway of 2 days to facilitate additional habituation sessions if necessary.

For acute pharmacological studies in a within‐subject design, length of testing is dependent on number of doses used. In the case of a 3‐dose study plus vehicle, 4 test sessions are necessary. Allowing for appropriate washout time between test sessions, a maximum of 2 sessions separated by 2 days a week is required. In this case, it would take 2 weeks to finish this dose response study. Dependent on choice of drug, multiple drugs may be tested in mice consecutively. Note that the number of behavioral runs is dependent on sample size and number of arenas available. In a typical set up, an *n* of 16 mice with 4 arenas is used. Two sets of 4 mice are run per day, meaning that a test session is split over two days. There is the option to increase number of arenas to reduce the number of behavioral runs; however, this is dependent on space and cost. An example timetable is provided in Figure 9.

If taking mice through the variations of the task (Alternate Protocols 1 and 2) as part of a chronic or phenotypic study, allow a period of 3 weeks including time for habituation. The effort curve paradigm requires 3 test sessions, with appropriate time between sessions of at least 1 day. The standard vs large forage arena paradigm requires 2 test sessions, with appropriate time between sessions of at least 1 day. An example timetable is provided in Figure 9.

### Author Contributions

**Eleanor Grayson**: Visualization; writing—original draft; writing—review and editing. **Foteini Xeni**: Data curation; formal analysis; investigation; writing—review and editing. **Caterina Marangoni**: Data curation; formal analysis; investigation. **Megan Jackson**: Conceptualization; data curation; formal analysis; funding acquisition; investigation; methodology; project administration; supervision; validation; visualization; writing—original draft; writing—review and editing.

### Conflict of Interest

The authors declare no conflict of interest.

## Data Availability

Data sharing is not applicable in this article.
